# Blood cytokine patterns suggest a modest inflammation phenotype in subjects with long‐chain fatty acid oxidation disorders

**DOI:** 10.14814/phy2.14037

**Published:** 2019-03-25

**Authors:** Colin S. McCoin, Melanie B. Gillingham, Trina A. Knotts, Jerry Vockley, Kikumi D. Ono‐Moore, Michael L. Blackburn, Jennifer E. Norman, Sean H. Adams

**Affiliations:** ^1^ Department of Molecular and Integrative Physiology Medical Center University of Kansas Kansas City Kansas; ^2^ Department of Molecular and Medical Genetics Oregon Health and Science University Portland Oregon; ^3^ School of Medicine Department of Anatomy, Physiology and Cell Biology University of California, Davis School of Veterinary Medicine Davis California; ^4^ Department of Pediatrics University of Pittsburgh Pittsburgh Pennsylvania; ^5^ Arkansas Children's Nutrition Center Little Rock Arkansas; ^6^ Department of Pediatrics University of Arkansas for Medical Sciences Little Rock Arkansas; ^7^ Department of Internal Medicine University of California Davis School of Medicine Davis California

**Keywords:** Carnitine, caspase‐3, immunometabolism

## Abstract

Excessive cellular accumulation or exposure to lipids such as long‐chain acylcarnitines (LCACs), ceramides, and others is implicated in cell stress and inflammation. Such a situation might manifest when there is a significant mismatch between long‐chain fatty acid (LCFA) availability versus storage and oxidative utilization; for example, in cardiac ischemia, increased LCACs may contribute to tissue cell stress and infarct damage. Perturbed LCFA *β*‐oxidation is also seen in fatty acid oxidation disorders (FAODs). FAODs typically manifest with fasting‐ or stress‐induced symptoms, and patients can manage many symptoms through control of diet and physical activity. However, episodic clinical events involving cardiac and skeletal muscle myopathies are common and can present without an obvious molecular trigger. We have speculated that systemic or tissue‐specific lipotoxicity and activation of inflammation pathways contribute to long‐chain FAOD pathophysiology. With this in mind, we characterized inflammatory phenotype (14 blood plasma cytokines) in resting, overnight‐fasted (~10 h), or exercise‐challenged subjects with clinically well‐controlled long‐chain FAODs (*n* = 12; 10 long‐chain 3‐hydroxyacyl‐CoA dehydrogenase [LCHAD]; 2 carnitine palmitoyltransferase 2 [CPT2]) compared to healthy controls (*n* = 12). Across experimental conditions, concentrations of three cytokines were modestly but significantly increased in FAOD (IFN *γ*, IL‐8, and MDC), and plasma levels of IL‐10 (considered an inflammation‐dampening cytokine) were significantly decreased. These novel results indicate that while asymptomatic FAOD patients do not display gross body‐wide inflammation even after moderate exercise, *β*‐oxidation deficiencies might be associated with chronic and subtle activation of “sterile inflammation.” Further studies are warranted to determine if inflammation is more apparent in poorly controlled long‐chain FAOD or when long‐chain FAOD‐associated symptoms are present.

## Introduction

Alterations in blood and tissue long‐chain acylcarnitines (LCACs, ≥C14 chain lengths) can occur through a number of physiological and pathophysiological metabolic events in which pools of acyl‐Coenzyme A fatty acid metabolites are converted to acylcarnitines through the actions of mitochondrial carnitine palmitoyltransferase 1 (CPT 1) and CPT 2. Both the quantity and pattern of LCACs are significantly altered in persons with inherited mitochondrial long‐chain fatty acid oxidation disorders (FAODs), the most common of which are CPT 2, very long chain acyl‐CoA dehydrogenase (VLCAD), and long‐chain 3‐hydroxyacyl‐CoA dehydrogenase (LCHAD) deficiencies. A proportion of the LCACs may then be exported and lead to higher plasma concentrations (e.g., see Wajner and Amaral 2015). Thus, plasma acylcarnitine patterns are utilized as a diagnostic marker of FAODs. In CPT2 deficiency, for example, blood LCAC concentrations (normally <1 *μ*mol/L) can reach ~5–45 *μ*mol/L (Isackson et al. 2008; McHugh et al. 2011).

More modest blood elevations of LCACs (and some medium‐chain acylcarnitines) are observed under conditions of insulin resistance and type 2 diabetes compared to insulin‐sensitive individuals (Inokuchi et al. 1995; Moder et al. 2003; Adams et al. 2009; Mihalik et al. 2010; Gillingham et al. 2013). A mismatch between LCFA availability and complete *β*‐oxidation is also inherent to exercise, during which LCFA metabolic flux is increased and blood concentrations of acylcarnitines reflective of incomplete FAO track muscle work (Lehmann et al. 2010; Zhang et al. 2017). Cardiac ischemia is another condition in which tissue LCACs and other lipids accumulate markedly, especially in heart regions with ischemic damage (Idell‐Wenger et al. 1978; Genuth and Hoppel 1981; Vogel‐van et al. 2011; Liepinsh et al. 2016). Myocellular concentrations of LCACs as estimated from wet weight vary widely in these dysmetabolic states, but range from >~50 *μ*mol/L (rodent insulin‐resistant muscle or LCFA‐treated cultured myocytes, Emerson et al. 2017; Koves et al. 2008), to >~1 mmol/L (rat or rabbit cardiac ischemia; i.e., Genuth and Hoppel 1981; Idell‐Wenger et al. 1978; Liepinsh et al. 2016).

Evidence has emerged that in addition to their utility as markers of metabolic disease and physiological shifts in LCFA metabolism, LCACs have bioactive properties that impinge upon multiple pathways involved in inflammation, cell stress, and insulin resistance (McCoin et al. 2015a). We have shown that exogenously applied LCAC (≥C14, starting at ~5–10 *μ*mol/L) activates, in a dose‐dependent fashion, cyclooxygenase‐2, TNF‐*α*, and MCP‐1 inflammatory cytokines in cultured murine RAW264.7 macrophage/monocytes (Rutkowsky et al. 2014); D,L‐C12‐ and D,L‐C14‐carnitine also activated an NF*κ*B‐reporter system (Adams et al. 2009). Pro‐inflammatory effects of C12‐carnitine in bone marrow‐derived macrophages were also reported, with the caveat that a cytotoxic concentration (100 *μ*mol/L) was tested (Sampey et al. 2012). Studies in murine C2C12 myotubes showed that palmitoylcarnitine increased muscle cell stress markers such as cell permeability, caspase‐3 cleavage, IL‐6 production, and MAP kinase pathways at concentrations of ≥~10–25 *μ*mol/L (McCoin et al. 2015b). The myocyte IL‐6 response was calcium‐dependent, and low concentrations of LCAC (5–10 *μ*mol/L) were found to trigger increases in intracellular calcium (McCoin et al. 2015b). Similar effects on IL‐6 secretion and cell stress were observed when PC3 prostate cancer cells were treated with 50 *μ*mol/L palmitoylcarnitine; intracellular calcium was increased in those cells at ≥5 *μ*mol/L (Al‐Bakheit et al. 2016).

Putative lipotoxic effects of excessive LCACs appear to manifest, in part, in mitochondria. In a cell model of inhibited diacylglycerol O‐acyltransferase 1‐dependent lipid droplet formation, nutrient deprivation elicited acylcarnitine accumulation coincident with a drop in mitochondrial membrane potential; the latter was rescued upon treatment of cells with the CPT1 inhibitor etomoxir (Nguyen et al. 2017). Concentrations of LCACs were increased by ~3‐fold in the mitochondrial membrane fraction of regions of rodent cardiac infarct damage, and application of 5‐10 *μ*mol/L palmitoylcarnitine to heart mitochondria elicited reduced oxidative phosphorylation (Liepinsh et al. 2016). Fibroblasts derived from patients with trifunctional protein deficiency (TFPD) displayed ~40% reduced O_2_ consumption in the presence of 200 *μ*mol/L palmitate, coincident with large increases in medium‐ to long‐chain acylcarnitines (Lefort et al. 2017). Treatment of TFPD fibroblasts with the CPT1 inhibitor etomoxir almost fully restored O_2_ consumption (Lefort et al. 2017). CPT1b‐/‐ mice were protected from muscle inflammation under modest fat feeding; in addition, CPT1b‐/‐ myotubes grown in the presence of 500 *μ*mol/L palmitate plus carnitine displayed >90% lower TNF*α* and IL6 mRNA levels compared to wild‐type cells (Warfel et al. 2016).

Taken together, these observations support the hypothesis that LCAC are bioactive lipids, and when in excess can drive cell stress outcomes and inflammation under some conditions. It is also possible that other lipotoxins and/or mitochondrial or cytosolic metabolic signals contribute to cell stress and inflammation when mitochondrial *β*‐oxidation is abnormal. For instance, the ceramide‐associated biochemical pathways (that utilize serine and palmitoyl‐CoA as initial substrates) give rise to lipid metabolites implicated in immunomodulatory and pro‐inflammation actions (e.g., ceramides, sphingosine‐1‐phosphate, ceramide‐1‐phosphate) (Hansen et al. 2015). Interestingly, the most significant blood metabolite signature shift in asymptomatic persons with long‐chain FAODs included complex lipids such as sphingomyelins, suggesting abnormal complex lipid metabolism concurrent with the well‐established elevations in blood LCACs (McCoin et al. 2016). With these issues in mind, we examined if the limited *β*‐oxidation associated with long‐chain FAOD translates into an increased inflammation phenotype. Blood plasma cytokine profiles in previously characterized FAOD subjects and healthy controls (McCoin et al. 2016) were compared, after a short fast and following a moderate exercise challenge. To our knowledge, this is the first study to examine inflammation phenotypes in individuals with inherited long‐chain FAODs.

## Materials and Methods

### Human subjects

Detailed information regarding the study and subject recruitment has been published previously (McCoin et al. 2016; Hait and Maiti 2017). Briefly, age‐, sex‐, and BMI‐matched long‐chain FAOD and control subjects were recruited to Oregon Health & Science University (OHSU) for a study approved by the OHSU Institutional Review Board (IRB no. 817). There were 12 subjects (seven male, five female) in the control group and 12 subjects in the FAOD (seven male, five female; two with CPT2 deficiency and 10 with LCHAD deficiency) (Table 1). FAOD was confirmed via medical record reviews and diagnostic evidence, except for one subject who did not present clinically with metabolic disease by traditional measures, but was diagnosed via genotype following diagnosis of a sibling's disease. All subjects were admitted to the OHSU Clinical and Translational Research Center for completion of the study procedures. FAOD subjects were all following a low‐fat diet upon admission (10% total energy from long‐chain triglycerides [LCT], 9–13% medium‐chain triglycerides [MCT], 56–76% carbohydrate, 8–15% protein), for 1.5 days prior to the fasting blood collection; 10 subjects were on prescribed carnitine supplementation (0.9 to 4 g per day), and no subjects were consuming triheptanoin. The control subjects were consuming their regular diet (approximately 31% energy from lipids [primarily LCTs], 10% from protein, and 59% from carbohydrates; self‐report). The participants underwent a 10‐h overnight fast after which blood was collected by venipuncture into EDTA vacutainer tubes, and plasma frozen at −80°C. Tetrahydrolipostatin was added to EDTA plasma to inhibit lipase hydrolysis of triglycerides. After fasting blood samples were obtained, subjects were fed a breakfast meal and 4 hr later a lunch meal. Meals for subjects with an FAOD were low‐fat (10% LCT), MCT supplemented (10% MCT) and provided 23‐30% of their estimated energy needs; control subjects consumed a normal fat meals (30% LCT with no MCT). An indwelling catheter was placed for repeated blood collection (typically in the antecubital vein). Two hours after lunch, 8 mL blood samples were drawn at pre‐exercise (0 min), immediate postexercise (40 min), and recovery (20 min after exercise cessation) time points. The moderate intensity treadmill exercise test has been described in detail (Behrend et al. 2012). Briefly, a 3‐min warm‐up phase with a slow walk at 1.5 miles per hour at 0% grade was followed by increases in rate and incline every 2 min until the subject's heart rate achieved 60–70% of his/her predicted maximum heart rate. Subjects were asked to continue exercising at 60–70% of their predicted maximum heart rate for an additional 40 min after the warm‐up phase.

**Table 1:** Participant characteristics for those with long‐chain fatty acid oxidation disorders and matched control subjects

	Subjects				Matched controls		
Dx	Mutation	Age (year)	Sex	BMI	Age (year)	Sex	BMI
LCHAD	c. 1528G>C/c.1528G>C	7	M	16.6	9	M	18.9
LCHAD	c. 1528G>C/c.2102A>G	7	M	19.2	8	M	15.3
LCHAD	c.1528G>C/c.1132C>T	8	M	18.8	10	M	15.1
LCHAD	c.1528G>C/exon 3 splice A + 3G	9	M	27.4	10	M	26.8
LCHAD	c. 1528G>C/c.1528G>C	14	M	19.3	15	M	19.7
LCHAD	c.1528G>C/c.274_278del	14	F	26.3	10	F	19.5
LCHAD	c.1528G>C/?	15	M	26.3	13	M	27.8
LCHAD	c.1528G>C/c.1678C>T	16	M	23.8	22	M	28.8
LCHAD	c. 1528G>C/c.1528C>T	16	F	22.6	17	F	21.2
CPT2	not detected^1^	16	F	22.7	16	F	22.1
LCHAD	c. 1528G>C/c.1528G>C	17	F	27.8	19	F	26.1
CPT2	not detected^1^	37	F	29.9	34	F	27.5

Dx, diagnosis; LCHAD, long‐chain 3‐hydroxy‐acyl‐CoA dehydrogenase deficiency; CPT2, carnitine palmitoyltransferase 2 deficiency; F, female; M, male; BMI, weight in kilograms/(height in meters)^2^

1 Common mutations p. 113S>L, p. 50P>H and p. 413 Q >fs were not detected; fs = frame shift; del = deletion.

Blood samples were stored in a study‐specific data repository and released with prior subject consent for this analysis (OHSU IRB 817). Fasting acylcarnitine profiles were provided in supplemental table 4 from our previous study (McCoin et al. 2016), and confirmed higher plasma long‐chain fatty acylcarnitine concentrations in the FAOD subjects.

### Cytokine analysis

Samples of 25 *μ*L of EDTA plasma per subject were assessed in duplicate for cytokine and chemokine concentrations using Meso Scale Discovery (MSD) Human Chemokine (K15001C‐1) and Human ProInflammatory (K15007C‐1) 9‐Plex Ultra Sensitive electrochemiluminescent Kits on the MSD Sector Imager 2400 following the manufacturer's instructions. Samples had one or two freeze–thaw cycles prior to cytokine analysis.

### Statistical analyses

Differences in plasma cytokines across treatment conditions were assessed using two‐way ANOVA, testing for effects of experimental condition, FAOD status, and experimental condition × FAOD status interactions (Graphpad Prism 6, Version 6.04). Determination of repeated measures two‐way ANOVA could not be routinely conducted due to occasional missing values arising from a lack of adequate archived sample to assay.

## Results

### Plasma cytokine patterns in healthy controls and FAOD subjects at rest and following exercise

**Figure 1:**
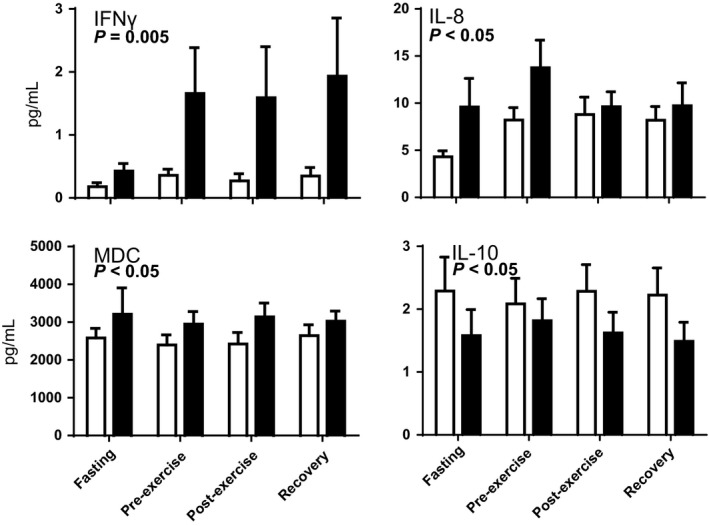
Blood plasma concentrations of cytokines that were significantly different when comparing healthy controls (*open bars, n *= 12) and subjects with long‐chain fatty acid oxidation disorders (FAODs)(*black bars, n *= 12). Blood was sampled after a brief overnight fast (~10 h, “Fasting”), just prior to an exercise bout (“Pre‐exercise,” 2 h post‐lunch), just after an exercise bout (“Postexercise,” ~5 min after cessation of exercise), and during recovery from the exercise bout (“Recovery,” 20 min after cessation of exercise, resting while sitting). Values are means ± SEM. Differences were evaluated with a two‐way ANOVA testing FAOD, test condition, and FAOD × test condition interactions; P‐values represent a significant FAOD effect. There was no significant effect of test condition and no significant FAOD × test condition interaction. *n* = 12 per group and test condition, except following removal of outliers: IFN *γ* (one control subject concentrations were an order of magnitude higher than all other subjects; one FAOD subject fasted value of 92 pg/mL).

**Figure 2:**
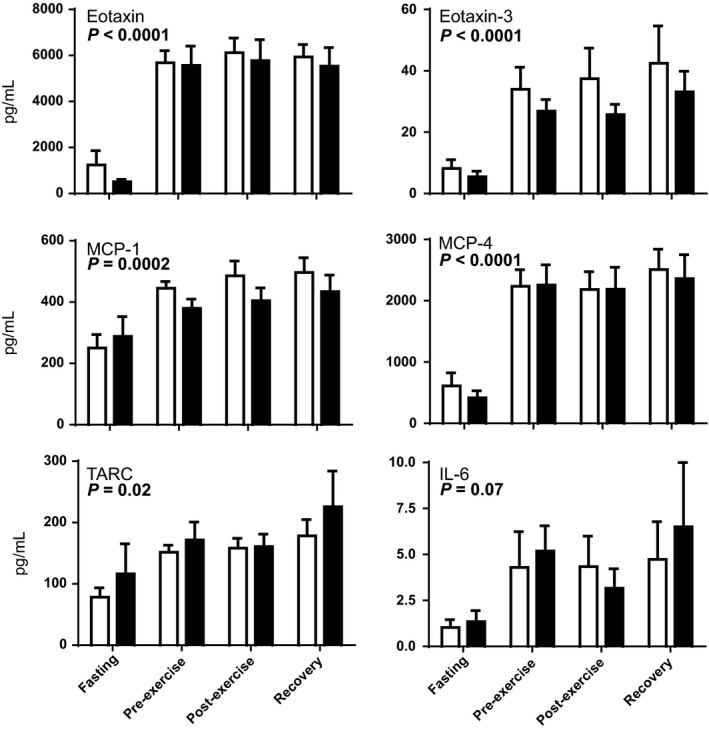
Blood plasma concentrations of cytokines that were significantly altered when comparing sampling condition (fasting versus other conditions) in healthy controls (*open bars, n *= 12) and subjects with long‐chain fatty acid oxidation disorders (FAODs) (*black bars, n *= 12). Blood was sampled after a brief overnight fast (~10 h, “Fasting”), just prior to an exercise bout (“Pre‐Exercise,” 2 h post‐lunch), just after an exercise bout (“Postexercise,” ~5 min after cessation of exercise), and during recovery from the exercise bout (“Recovery,” 20 min after cessation of exercise, resting while sitting). Values are means ± SEM. Differences were evaluated with a two‐way ANOVA testing FAOD, test condition, and FAOD x test condition interactions; *P*‐values represent a significant test condition effect. There was no significant effect of FAOD and no significant FAOD x test condition interaction. *n* = 12 per group and test condition, except following removal of outliers: TARC (one control subject and one FAOD subject fasting values were 897 and 1226 pg/mL, respectively).

**Figure 3:**
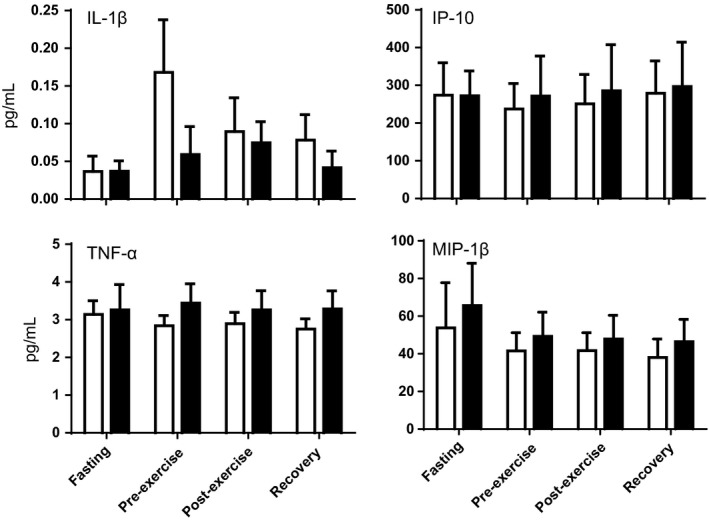
Blood plasma concentrations of cytokines that were not significantly altered by sampling condition or FAOD status in healthy controls (*open bars, n *= 12) and subjects with long‐chain fatty acid oxidation disorders (FAODs) (*black bars, n *= 12). Blood was sampled after a brief overnight fast (~10 h, “Fasting”), just prior to an exercise bout (“Pre‐Exercise,” 2 h post‐lunch), just after an exercise bout (“Postexercise,” ~5 min after cessation of exercise), and during recovery from the exercise bout (“Recovery,” 20 min after cessation of exercise, resting while sitting). Values are means ± SEM. Differences were evaluated with a two‐way ANOVA testing FAOD, test condition, and FAOD x test condition interactions. *n* = 12 per group and test condition, except following removal of outliers: IL‐1*β* (two FAOD subject values [pre‐exercise, postexercise] that were an order of magnitude higher than all other values).

To determine if long‐chain FAOD leads to inherently stimulated systemic inflammation, concentrations of a panel of 15 plasma cytokines were determined following an overnight fast, at rest just prior to exercise (~2 h post‐lunch), just after exercise, and then following 20 min of postexercise recovery (Figs. 1, 2, 3). Despite significant person‐to‐person variability in cytokine concentrations, three major findings were observed. First, regardless of test condition, subjects with FAOD showed modestly increased markers of inflammation, including significantly higher plasma concentrations of IFN‐*γ*, IL‐8, and MDC (a.k.a. CCL22) (Fig. 1). Plasma IL‐10, a canonical “inflammation‐dampening” cytokine, was modestly lower in the FAOD participants (Fig. 1). Second, some plasma cytokines were influenced by test condition in that they were significantly higher in the pre‐exercise, postexercise, and recovery periods relative to the ~10 h fasted state, regardless of FAOD status: eotaxin, eotaxin‐3, MCP‐1, MCP‐4, and TARC (Fig. 2). Plasma IL‐6 levels in the pre‐exercise, postexercise, and recovery time points trended higher compared to fasting, but this effect was not statistically significant as determined by a standard two‐way ANOVA (*P* = 0.07, time effect) (Fig. 2). Plasma concentrations for IL‐1*β*, TNF‐*α*, IP‐10, and MIP‐1*β* were not impacted significantly by FAOD or the experimental conditions (Fig. 3).

## Discussion

Abnormally high accumulation of metabolites associated with inefficient or incomplete mitochondrial LCFA *β*‐oxidation, including LCACs and certain complex lipids, has been proposed to contribute to inflammation and cellular dysfunction (see Hansen et al. 2015; McCoin et al. 2015a). Moderate to severe “lipotoxicity” could in theory impact outcomes as disparate as insulin resistance, cardiac ischemia injury, and myopathies, by triggering cell stress and inflammation pathways. For instance, we and others have described a variety of insulin resistance, cell stress, and pro‐inflammatory responses to experimental provision of LCACs, using myocytes, monocyte‐macrophages, or isolated mitochondrial preparations (Adams et al. 2009; Sampey et al. 2012; Rutkowsky et al. 2014; Aguer et al. 2015; Al‐Bakheit et al. 2016). Diminution of LCAC production in myocytes, heart or muscle tissue, through treatment with 2‐tetradecylglycidic acid (TDGA) CPT1 inhibitor (Vogel‐van et al. 2011), 4‐[ethyl(dimethyl)ammonio] butanoate (methyl‐GBB) or mildronate OCT2 carnitine transporter inhibitors (Liepinsh et al. 2014, 2016; Dambrova et al. 2016), and genetic ablation of muscle CPT1b (Warfel et al. 2016) tends to rescue or normalize cell stress‐inflammation phenotypes that accompany inefficient *β*‐oxidation or experimental provision of LCFA. These observations highlight excessive LCACs as potential lipotoxins; however, mismatched LCFA availability relative to mitochondrial fatty acid oxidation would increase cellular concentrations of many other lipid derivatives upstream of CPT1, and alter non‐lipid metabolite pools as well. One or more of these events could promote cell stress and trigger inflammation‐associated pathways.

In patients with long‐chain FAODs, the potential for metabolite toxicity to cause or exacerbate clinical episodes such as cardiac and skeletal muscle myopathy remains underexplored, and specific molecular factors that link altered lipid metabolism to clinical (and subclinical) phenomena remain largely unknown. Using archived samples collected from a previous study, we had an opportunity to examine, for the first time, systemic inflammation markers in long‐chain FAOD patients as compared to controls at rest and following an exercise challenge. We reasoned that the latter condition could unveil otherwise subtle inflammation phenotypes in FAOD, since exercise drives LCFA mobilization (Hekimian and Feuvray 1986; Romijn et al. 1993; Brooks 1997) and hence would promote production of LCACs and other lipid derivatives. The results indicate that in clinically well‐controlled, asymptomatic FAOD participants, body‐wide impairment of *β*‐oxidation does not inherently lead to gross inflammation outcomes as measured by comprehensive blood cytokine profiling. Nevertheless, there were modest changes in select cytokines reflective of subclinical activation of “sterile inflammation” (inflammation induced from non‐pathogen triggers such as metabolites). For example, small but statistically significant increases were observed for plasma IFN*γ*, IL‐8, and MDC in persons with FAOD, along with significantly lower concentrations of IL‐10. These subtle shifts in cytokines may point to chronic activation of inflammation associated with metabolic perturbations, at least in a subset of immune cells or tissues. Considering the small sample sizes available for controlled studies of rare long‐chain FAODs, and the high person‐to‐person variability in blood cytokine levels, it will be important to validate the current results in additional cohorts and alternative experimental paradigms. For instance, future studies could explore whether cells isolated from FAOD patients display higher sensitivity in terms of inflammation or cell stress in response to LCFA or other challenges. The FAOD participants in the study were clinically well; it is possible that FAOD‐associated inflammation becomes more pronounced under metabolically stressed conditions, leading to skeletal muscle or cardiac symptoms. In other words, lipotoxemia‐associated inflammation may only manifest with a “multiple hit.” This question can be explored by comparing cytokine patterns alongside LCACs, LCFAs, and complex lipids in persons with FAOD in the asymptomatic state versus when they present with clinical symptoms (e.g., myopathy and rhabdomyolysis). It may also be of interest to explore inflammation phenotypes in other inherited disorders of metabolism in which mitochondrial fuel oxidation is disrupted.

An interesting and unanticipated observation in the current study was that independent of FAOD status, several plasma cytokines rose significantly in the pre‐exercise to recovery phase of the study when compared to the overnight‐fasted state. From our results, we cannot conclude what factors drove these outcomes. However, a possible explanation is that transitioning to the fed state triggered a normal postprandial inflammation response (breakfast and lunch were provided in the hours leading up to collection of the “Pre‐Exercise” sample). Postprandial inflammation has been reported by many investigators, although these observations are not universal and postprandial phenotypes may be nutrient‐ and cytokine‐specific (see Ford et al. 1996). No matter what post‐fasting events (meal‐dependent or meal‐independent) altered cytokine levels, the lack of an FAOD effect *per se* on the post‐fasting increases in cytokines suggests that signals emanating from mitochondrial LCFA *β*‐oxidation were not involved.

In conclusion, a deficiency in mitochondrial catabolism of LCFA in clinically stable, asymptomatic patients with FAODs does not lead to marked systemic inflammation. However, modest changes in select cytokines are consistent with the hypothesis that chronic, subclinical “sterile inflammation” is associated with long‐chain FAOD. It is acknowledged that observations herein are limited to a small number of clinically well‐managed patients, and in whom typical dietary patterns were quite different when compared to control participants (e.g., more MCT and high carbohydrates in prescribed FAOD diets). It should also be noted that recruiting large numbers of age‐ and sex‐matched individuals with these rare long‐chain FAODs is a major challenge, and the cohort herein was quite broad in age which may have contributed to variance. The current paper reflects a secondary analysis samples from a previously described cohort, and hence we did not have an opportunity to recruit prospectively for this specific line of research. Thus, additional controlled experiments and clinical studies are warranted to determine if inflammation phenotypes precede or are coincident with the episodic symptoms common to long‐chain FAOD.

## Conflict of Interest

None declared.

## Informed Consent

All procedures followed were in accordance with the ethical standards of the responsible committee on human experimentation (institutional and national) and with the Helsinki Declaration of 1975, as revised in 2000. Written informed consent was obtained from all patients or legal guardians prior to inclusion in the study.

## References

[phy214037-bib-0001] Adams, S. H. , C. L. Hoppel , K. H. Lok , L. Zhao , S. W. Wong , P. E. Minkler , et al. 2009 Plasma acylcarnitine profiles suggest incomplete long‐chain fatty acid beta‐oxidation and altered tricarboxylic acid cycle activity in type 2 diabetic African‐American women. J. Nutr. 139:1073–1081. 19369366 10.3945/jn.108.103754 PMC2714383

[phy214037-bib-0002] Aguer, C. , C. S. McCoin , T. A. Knotts , A. B. Thrush , K. Ono‐Moore , R. McPherson , et al. 2015 Acylcarnitines: potential implications for skeletal muscle insulin resistance. FASEB J. 29:336–345. 25342132 10.1096/fj.14-255901 PMC4285541

[phy214037-bib-0003] Al‐Bakheit, A. , M. Traka , S. Saha , R. Mithen , and A. Melchini . 2016 Accumulation of palmitoylcarnitine and its effect on pro‐inflammatory pathways and calcium influx in prostate cancer. Prostate 76:1326–1337. 27403764 10.1002/pros.23222 PMC4996340

[phy214037-bib-0004] Behrend, A. M. , C. O. Harding , J. D. Shoemaker , D. Matern , D. J. Sahn , D. L. Elliot , et al. 2012 Substrate oxidation and cardiac performance during exercise in disorders of long chain fatty acid oxidation. Mol. Genet. Metab. 105:110–115. 22030098 10.1016/j.ymgme.2011.09.030 PMC3253922

[phy214037-bib-0005] Brooks, G. A. 1997 Importance of the ‘crossover' concept in exercise metabolism. Clin. Exp. Pharmacol. Physiol. 24:889–895. 9363377 10.1111/j.1440-1681.1997.tb02712.x

[phy214037-bib-0006] Dambrova, M. , M. Makrecka‐Kuka , R. Vilskersts , E. Makarova , J. Kuka , and E. Liepinsh . 2016 Pharmacological effects of meldonium: biochemical mechanisms and biomarkers of cardiometabolic activity. Pharmacol. Res. 113:771–780. 26850121 10.1016/j.phrs.2016.01.019

[phy214037-bib-0007] Emerson, S. R. , S. P. Kurti , C. A. Harms , M. D. Haub , T. Melgarejo , C. Logan , et al. 2017 Magnitude and timing of the postprandial inflammatory response to a high‐fat meal in healthy adults: a systematic review. Adv. Nutr. 8:213–225. 28298267 10.3945/an.116.014431 PMC5347112

[phy214037-bib-0008] Ford, D. A. , X. Han , C. C. Horner , and R. W. Gross . 1996 Accumulation of unsaturated acylcarnitine molecular species during acute myocardial ischemia: metabolic compartmentalization of products of fatty acyl chain elongation in the acylcarnitine pool. Biochemistry 35:7903–7909. 8672492 10.1021/bi960552n

[phy214037-bib-0009] Genuth, S. M. , and C. L. Hoppel . 1981 Acute hormonal effects on carnitine metabolism in thin and obese subjects: responses to somatostatin, glucagon, and insulin. Metabolism 30:393–401. 6111018 10.1016/0026-0495(81)90121-9

[phy214037-bib-0010] Gillingham, M. B. , C. O. Harding , D. A. Schoeller , D. Matern , and J. Q. Purnell . 2013 Altered body composition and energy expenditure but normal glucose tolerance among humans with a long‐chain fatty acid oxidation disorder. Am. J. Physiol. Endocrinol. Metab. 305:E1299–E1308. 24064340 10.1152/ajpendo.00225.2013 PMC3840216

[phy214037-bib-0011] Hait, N. C. , and A. Maiti . 2017 The role of sphingosine‐1‐phosphate and ceramide‐1‐phosphate in inflammation and cancer. Mediators Inflamm. 2017:4806541. 29269995 10.1155/2017/4806541 PMC5705877

[phy214037-bib-0012] Hansen, J. S. , X. Zhao , M. Irmler , X. Liu , M. Hoene , M. Scheler , et al. 2015 Type 2 diabetes alters metabolic and transcriptional signatures of glucose and amino acid metabolism during exercise and recovery. Diabetologia 58:1845–1854. 26067360 10.1007/s00125-015-3584-x

[phy214037-bib-0013] Hekimian, G. , and D. Feuvray . 1986 Reduction of ischemia‐induced acyl carnitine accumulation by TDGA and its influence on lactate dehydrogenase release in diabetic rat hearts. Diabetes 35:906–910. 3732631 10.2337/diab.35.8.906

[phy214037-bib-0014] Idell‐Wenger, J. A. , L. W. Grotyohann , and J. R. Neely . 1978 Coenzyme A and carnitine distribution in normal and ischemic hearts. J. Biol. Chem. 253:4310–4318. 207696

[phy214037-bib-0015] Inokuchi, T. , K. Imamura , K. Nomura , K. Nomoto , and S. Isogai . 1995 Changes in carnitine metabolism with ketone body production in obese glucose‐intolerant patients. Diabetes Res. Clin. Pract. 30:1–7. 8745200 10.1016/0168-8227(95)01140-4

[phy214037-bib-0016] Isackson, P. J. , M. J. Bennett , U. Lichter‐Konecki , M. Willis , W. L. Nyhan , V. R. Sutton , et al. 2008 CPT2 gene mutations resulting in lethal neonatal or severe infantile carnitine palmitoyltransferase II deficiency. Mol. Genet. Metab. 94:422–427. 18550408 10.1016/j.ymgme.2008.05.002

[phy214037-bib-0017] Koves, T. R. , J. R. Ussher , R. C. Noland , D. Slentz , M. Mosedale , O. Ilkayeva , et al. 2008 Mitochondrial overload and incomplete fatty acid oxidation contribute to skeletal muscle insulin resistance. Cell Metab. 7:45–56. 18177724 10.1016/j.cmet.2007.10.013

[phy214037-bib-0018] Lefort, B. , E. Gouache , C. Acquaviva , M. Tardieu , J. F. Benoist , J. F. Dumas , et al. 2017 Pharmacological inhibition of carnitine palmitoyltransferase 1 restores mitochondrial oxidative phosphorylation in human trifunctional protein deficient fibroblasts. Biochim. Biophys. Acta 1863:1292–1299. 10.1016/j.bbadis.2017.04.005 28392417

[phy214037-bib-0019] Lehmann, R. , X. Zhao , C. Weigert , P. Simon , E. Fehrenbach , J. Fritsche , et al. 2010 Medium chain acylcarnitines dominate the metabolite pattern in humans under moderate intensity exercise and support lipid oxidation. PLoS ONE 5:e11519. 20634953 10.1371/journal.pone.0011519 PMC2902514

[phy214037-bib-0020] Liepinsh, E. , M. Makrecka , J. Kuka , H. Cirule , E. Makarova , E. Sevostjanovs , et al. 2014 Selective inhibition of OCTN2 is more effective than inhibition of gamma‐butyrobetaine dioxygenase to decrease the availability of l‐carnitine and to reduce myocardial infarct size. Pharmacol. Res. 85:33–38. 24836867 10.1016/j.phrs.2014.05.002

[phy214037-bib-0021] Liepinsh, E. , M. Makrecka‐Kuka , K. Volska , J. Kuka , E. Makarova , U. Antone , et al. 2016 Long‐chain acylcarnitines determine ischaemia/reperfusion‐induced damage in heart mitochondria. Biochem J. 473:1191–1202. 26936967 10.1042/BCJ20160164

[phy214037-bib-0022] McCoin, C. S. , T. A. Knotts , and S. H. Adams . 2015a Acylcarnitines–old actors auditioning for new roles in metabolic physiology. Nat. Rev. Endocrinol. 11:617–625. 26303601 10.1038/nrendo.2015.129 PMC4966159

[phy214037-bib-0023] McCoin, C. S. , T. A. Knotts , K. D. Ono‐Moore , P. J. Oort , and S. H. Adams . 2015b Long‐chain acylcarnitines activate cell stress and myokine release in C2C12 myotubes: calcium‐dependent and ‐independent effects. Am. J. Physiol. Endocrinol. Metab. 308:E990–E1000. 25852008 10.1152/ajpendo.00602.2014 PMC4451287

[phy214037-bib-0024] McCoin, C. S. , B. D. Piccolo , T. A. Knotts , D. Matern , J. Vockley , M. B. Gillingham , et al. 2016 Unique plasma metabolomic signatures of individuals with inherited disorders of long‐chain fatty acid oxidation. J. Inherit. Metab. Dis. 39:399–408. 26907176 10.1007/s10545-016-9915-3 PMC4851894

[phy214037-bib-0025] McHugh, D. , C. A. Cameron , J. E. Abdenur , M. Abdulrahman , O. Adair , S. A. Al Nuaimi , et al. 2011 Clinical validation of cutoff target ranges in newborn screening of metabolic disorders by tandem mass spectrometry: a worldwide collaborative project. Genet. Med. 13:230–254. 21325949 10.1097/GIM.0b013e31820d5e67

[phy214037-bib-0026] Mihalik, S. J. , B. H. Goodpaster , D. E. Kelley , D. H. Chace , J. Vockley , F. G. Toledo , et al. 2010 Increased levels of plasma acylcarnitines in obesity and type 2 diabetes and identification of a marker of glucolipotoxicity. Obesity (Silver Spring) 18:1695–1700. 20111019 10.1038/oby.2009.510 PMC3984458

[phy214037-bib-0027] Moder, M. , A. Kiessling , H. Loster , and L. Bruggemann . 2003 The pattern of urinary acylcarnitines determined by electrospray mass spectrometry: a new tool in the diagnosis of diabetes mellitus. Anal. Bioanal. Chem. 375:200–210. 12560963 10.1007/s00216-002-1654-7

[phy214037-bib-0028] Nguyen, T. B. , S. M. Louie , J. R. Daniele , Q. Tran , A. Dillin , R. Zoncu , et al. 2017 DGAT1‐dependent lipid droplet biogenesis protects mitochondrial function during starvation‐induced autophagy. Dev. Cell 42:9–21 e25. 28697336 10.1016/j.devcel.2017.06.003 PMC5553613

[phy214037-bib-0029] Romijn, J. A. , E. F. Coyle , L. S. Sidossis , A. Gastaldelli , J. F. Horowitz , E. Endert , et al. 1993 Regulation of endogenous fat and carbohydrate metabolism in relation to exercise intensity and duration. Am. J. Physiol. 265:E380–E391. 8214047 10.1152/ajpendo.1993.265.3.E380

[phy214037-bib-0030] Rutkowsky, J. M. , T. A. Knotts , K. D. Ono‐Moore , C. S. McCoin , S. Huang , D. Schneider , et al. 2014 Acylcarnitines activate proinflammatory signaling pathways. Am. J. Physiol. Endocrinol. Metab. 306:E1378–E1387. 24760988 10.1152/ajpendo.00656.2013 PMC4059985

[phy214037-bib-0031] Sampey, B. P. , A. J. Freemerman , J. Zhang , P. F. Kuan , J. A. Galanko , T. M. O'Connell , et al. 2012 Metabolomic profiling reveals mitochondrial‐derived lipid biomarkers that drive obesity‐associated inflammation. PLoS ONE 7:e38812. 22701716 10.1371/journal.pone.0038812 PMC3373493

[phy214037-bib-0032] Vogel‐van, De , J. den Bosch , J. Hoeks , S. Timmers , S. M. Houten , P. J. van Dijk , et al. 2011 The effects of long‐ or medium‐chain fat diets on glucose tolerance and myocellular content of lipid intermediates in rats. Obesity (Silver Spring) 19:792–799. 20595951 10.1038/oby.2010.152

[phy214037-bib-0033] Wajner, M. , and A. U. Amaral . 2015 Mitochondrial dysfunction in fatty acid oxidation disorders: insights from human and animal studies. Biosci. Rep. 36:e00281. 26589966 10.1042/BSR20150240 PMC4718505

[phy214037-bib-0034] Warfel, J. D. , E. M. Bermudez , T. M. Mendoza , S. Ghosh , J. Zhang , C. M. Elks , et al. 2016 Mitochondrial fat oxidation is essential for lipid‐induced inflammation in skeletal muscle in mice. Sci. Rep. 6:37941. 27892502 10.1038/srep37941 PMC5124994

[phy214037-bib-0035] Zhang, J. , A. R. Light , C. L. Hoppel , C. Campbell , C. J. Chandler , D. J. Burnett , et al. 2017 Acylcarnitines as markers of exercise‐associated fuel partitioning, xenometabolism, and potential signals to muscle afferent neurons. Exp. Physiol. 102:48–69. 27730694 10.1113/EP086019 PMC5209287

